# Editorial Note: Analysis of the importance and priority of male and female players in mixed doubles table tennis

**DOI:** 10.1371/journal.pone.0357531

**Published:** 2026-09-03

**Authors:** The PLOS One Editors

The *PLOS One* Editors issue this Editorial Note to inform readers that works cited in this article [1] as References 8 and 23 were retracted before the article’s publication date. PLOS considers that these references are not crucial in supporting the research or conclusions reported in [1].
